# Airway stenosis alters the balance between tidal volume, air entrainment, and oxygen-source delivery during normal frequency and superimposed jet ventilation: a bench model study

**DOI:** 10.3389/fmed.2026.1910316

**Published:** 2026-10-28

**Authors:** Mingyuan Yang, Zhuomin Deng, Hong Li, Jing Guo, Yuxue Yao, Shuwang Yang, Qinghao Cheng

**Affiliations:** 1 Department of Anesthesiology, Emergency General Hospital, Beijing, China; 2 PMLS Upstream Marketing Department, Shenzhen Mindray Bio-medical Electronics Co., Ltd, Shenzhen, China

**Keywords:** air entrainment, airway stenosis, jet ventilation, normal frequency jet ventilation, oxygen concentration, rigid bronchoscopy, superimposed high-frequency jet ventilation, tidal volume

## Abstract

**Background:**

Severe airway stenosis alters gas mixing and volume delivery during open-system jet ventilation. How high-frequency (HF) superimposition and component titration modify these effects remains incompletely characterized.

**Objective:**

To compare stenosis-induced changes in distal oxygen concentration (FiO_2_), tidal volume (VT), pressure, flow, and estimated gas composition among pure normal-frequency jet ventilation (NFJV) and superimposed HF jet ventilation (SHFJV) with titration of either the NF (SHFJV-NF) or HF component (SHFJV-HF).

**Methods:**

An open rigid-bronchoscopy bench model was tested with and without 90% fixed airway stenosis. Pure NFJV and SHFJV-NF shared 56 NF parameter settings; SHFJV-HF included 60 HF settings with a fixed NF component. Linear mixed-effects models evaluated strategy × airway interactions and Holm-adjusted pairwise contrasts. A separate matched model assessed HF addition. Gas-component fractions and volumes were estimated at each setting using a two-source mixing model.

**Results:**

Strategy × airway interactions were significant for all five measured outcomes (all *P* < 0.001). Median stenosis-induced changes were +4.7, +29.1, and +24.7 percentage points for FiO_2_ and −454, −577, and −232 mL for VT during pure NFJV, SHFJV-NF, and SHFJV-HF, respectively. At identical NF settings, HF addition modified the stenosis response by +24.1 percentage points for FiO_2_, −111.7 mL for VT (95% CI −173.6 to −49.8), +2.02 cmH_2_O for measured positive end-expiratory pressure (PEEP), and −8.11 L·min^−1^ for expiratory flow magnitude (all *P* < 0.001). Estimated entrained-air volume decreased in all strategies, whereas oxygen-source volume did not increase uniformly.

**Conclusion:**

At identical NF settings, HF addition amplified the stenosis-associated FiO_2_ rise, VT loss, and PEEP increase. HF-component titration produced a different response across its tested parameter range. Estimated gas-composition changes were consistent with reduced oxygen dilution. Distal FiO_2_ did not establish preserved ventilation or arterial oxygenation, and CO_2_ elimination was not measured.

## Introduction

Jet ventilation is widely used during rigid bronchoscopy and airway interventions because it can maintain gas delivery while preserving procedural access through an open airway. Available ventilation approaches during rigid bronchoscopy include apneic oxygenation, spontaneous or controlled ventilation, manual or normal-frequency jet ventilation (NFJV), high-frequency jet ventilation (HFJV), and superimposed high-frequency jet ventilation (SHFJV) (1, 2). Unlike conventional ventilation through a sealed circuit, jet ventilation during rigid bronchoscopy is usually delivered through an uncuffed and open system. In this setting, the gas entering the distal airway is not determined only by the jet source, but also by airway geometry, jet-induced entrainment of ambient air, mixing within the airway, and expiratory egress.

NFJV provides intermittent convective gas delivery at frequencies close to physiological respiratory rates. In clinical practice, this mode is often used during rigid bronchoscopy because it can generate visible chest movement and relatively large tidal excursions while maintaining an open airway. However, the delivered tidal volume during NFJV is not directly set by the ventilator. Instead, it is influenced by driving pressure, inspiratory time, airway resistance, lung mechanics, and the degree of ambient-air entrainment. Our previous bench work in a normal rigid bronchoscopy model showed that jet ventilation mode, driving pressure, and frequency substantially affect tidal volume, airway pressure, and flow dynamics during NFJV, HFJV, and SHFJ V (3). These findings indicate that even in a non-stenotic open airway, the relationship between jet settings and delivered gas is complex.

Airway stenosis further modifies this relationship. Previous bench and experimental studies had shown that upper airway narrowing can alter air entrainment, airway pressure transmission, and expiratory emptying during jet ventilation (4–6). Buczkowski et al. demonstrated that air entrainment during HFJV is affected by upper tracheal stenosis, supporting the concept that stenosis can change the composition of gas delivered downstream (4). Our subsequent bench study showed that severe fixed stenosis reduced tidal volume (VT) and expiratory flow while increasing measured end-expiratory pressure and distal oxygen concentration during single-frequency jet ventilation (7). These findings establish that the effect of narrowing depends on the delivery configuration and that a high distal oxygen concentration (FiO_2_) can coexist with impaired volume delivery.

SHFJV combines a normal-frequency component with a high-frequency component. Clinical and experimental studies suggest that SHFJV can support ventilation during endoscopic laryngotracheal surgery and may influence lung volume and gas exchange differently from single-frequency jet ventilation (8–10). Because SHFJV includes both a convective normal-frequency component and a high-frequency component, it may also change the balance between total delivered volume and jet-induced entrainment in an open airway. Whether this balance is preserved or altered when severe airway stenosis limits entrainment and expiratory egress remains unclear.

We therefore compared paired responses to 90% fixed airway stenosis during pure NFJV, SHFJV with NF-component titration (SHFJV-NF), and SHFJV with HF-component titration (SHFJV-HF). We also compared pure NFJV and SHFJV-NF at identical NF settings. We hypothesized that HF addition and component titration would modify the magnitude of stenosis-induced changes in distal FiO_2_, VT, pressure, flow, and estimated gas composition.

## Methods

### Study design

This bench study used the open rigid-bronchoscopy platform described previously (3, 7). Measurements for the present study were obtained in experimental sessions separate from those of our previous studies. Three ventilation strategies were evaluated under non-stenotic and 90% stenotic airway conditions. The study involved no human participants, animals, or biological specimens. The protocol was reviewed and approved as exempt by the Academic Ethics Committee of Emergency General Hospital, Beijing, China (Protocol No. KY202521); informed consent was not applicable.

### Airway models and experimental setup

Two adult airway configurations were evaluated: a non-stenotic airway model and a fixed severe stenosis model. The non-stenotic airway model had an internal diameter of 15 mm. The stenotic model contained a fixed circumferential narrowing corresponding to 90% reduction in cross-sectional area. The narrowed segment was located in the mid-portion of the airway model, whereas the proximal and distal airway segments retained the same dimensions as the non-stenotic model.

The experimental circuit consisted of a jet ventilator, a rigid bronchoscope sheath, the 3D-printed airway model, a flow analyzer, and an artificial test lung. Jet ventilation was delivered using a Mindray A7 anesthesia workstation (Mindray Bio-Medical Electronics Co., Ltd., Shenzhen, China). The rigid bronchoscope sheath was compatible with a size 10# rigid bronchoscope (Karl Storz, Tuttlingen, Germany). The distal end of the airway model was connected in series to a flow analyzer (PF-300 PRO, IMT Analytics, Switzerland) and an artificial test lung (Michigan Instruments, USA). The physical experimental setup is shown in Figure 1.

**Figure 1:**
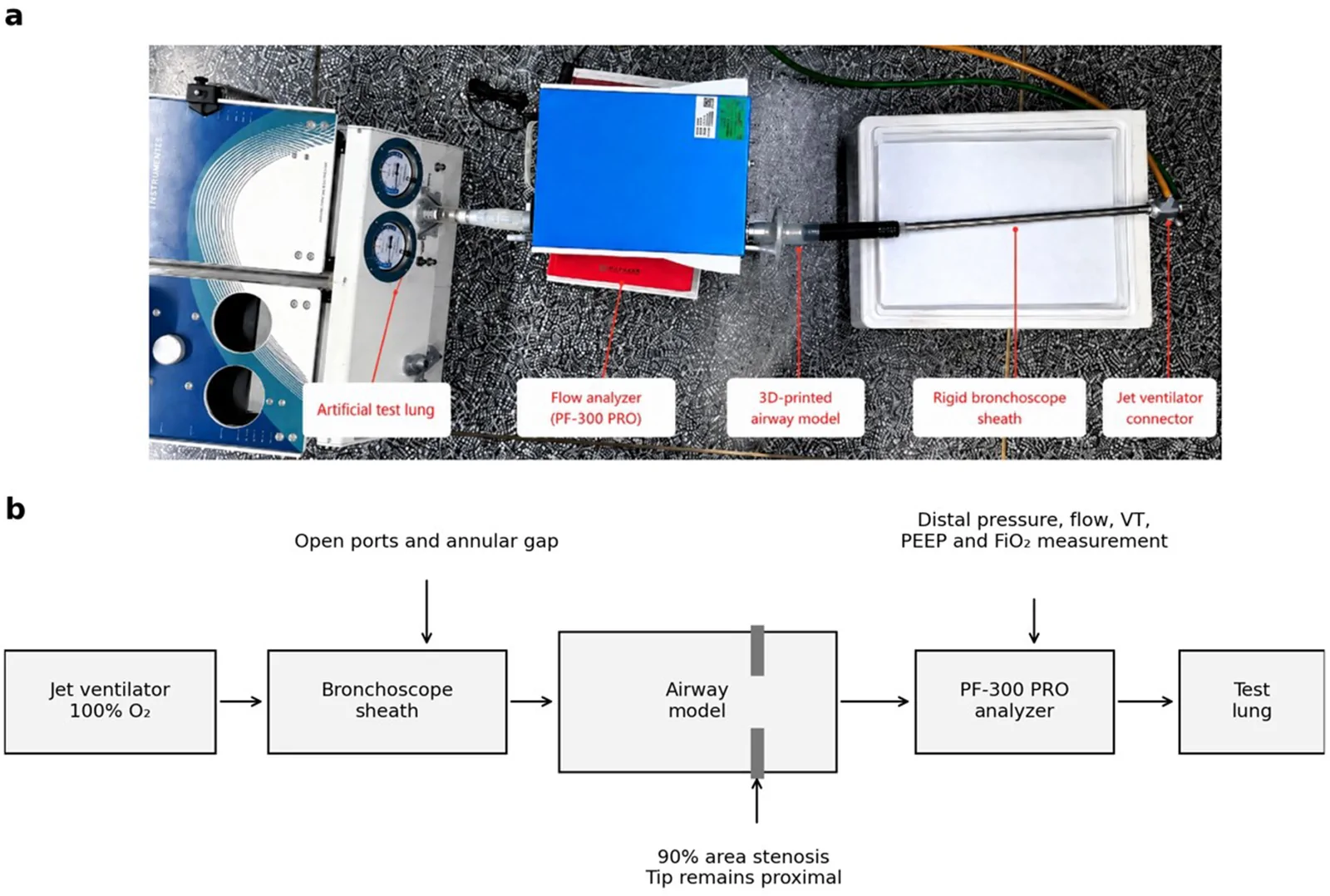
Experimental setup and measurement configuration. **(a)** Photograph of the rigid-bronchoscopy bench platform, showing the artificial test lung, PF- 300 PRO flow analyzer, 3D-printed airway model, rigid bronchoscope sheath, and jet connector. Reproduced from “Physical laboratory setup of the rigid bronchoscopy jet ventilation bench model” by Mingyuan Yang, Zhuomin Deng, Hong Li, Jing Guo, Yuxue Yao, Shuwang Yang and Qinghao Cheng, licensed under CC BY 4.0. **(b)** Schematic of gas delivery and distal measurement. The bronchoscope tip remained proximal to the fixed stenosis, which reduced cross-sectional area by 90%. The analyzer measured pressure, flow, VT, PEEP, and mixed-gas FiO_2_ upstream of the test lung. Ventilator-set driving pressure is distinct from measured distal airway pressure. The schematic is not to scale.

The rigid bronchoscope sheath was inserted into the proximal end of the airway model to reproduce the open mechanical configuration of rigid bronchoscopy. In this configuration, the sheath occupied part of the airway lumen but did not completely seal the airway. The remaining annular space between the sheath and the airway wall allowed ambient-air entrainment and expiratory gas egress. The bronchoscope tip position was kept constant between airway models. In the stenotic model, the tip was positioned proximal to the narrowed segment. In the non-stenotic model, the same axial location was used.

Airway pressure, flow, tidal volume, PEEP, and FiO_2_ were recorded by the flow analyzer at the distal airway, immediately upstream of the artificial test lung. This measurement position was used to characterize the mixed gas entering the test lung. The artificial lung settings were kept constant throughout all experiments. The delivered oxygen fraction from the jet source was set to 1.0 for all ventilation strategies. The inspiratory-to-expiratory ratio was fixed at 1:1.

Ventilator-set driving pressure was expressed in bar (1 bar = 100 kPa), as displayed by the device. This setting describes the upstream jet supply and is distinct from pressure measured in the distal airway, which was expressed in cmH_2_O. No direct measurement of nozzle exit pressure was performed. PEEP denotes the analyzer-recorded end-expiratory pressure; it was not a direct measurement of regional alveolar intrinsic PEEP.

### Ventilation strategies and parameter settings

Three jet ventilation strategies were evaluated. Pressure and frequency ranges covered settings used in our practice and adjacent settings selected to characterize the mechanical response. The tested combinations were experimental settings rather than a validated clinical operating range.

First, pure normal-frequency jet ventilation was tested as a single normal-frequency jet mode and is referred to as pure NFJV. In this strategy, the jet driving pressure was tested at 0.7, 0.9, 1.1, 1.3, 1.5, 1.7, 1.9, and 2.0 bar. The ventilation frequency was tested at 12, 14, 16, 18, 20, 22, and 24 cycles·min^−1^. This generated 56 parameter combinations for paired airway comparison.

Second, superimposed high-frequency jet ventilation with normal-frequency component titration was tested and is referred to as SHFJV-NF. In this strategy, the high-frequency component was kept constant at a driving pressure of 0.6 bar and a frequency of 600 cycles·min^−1^. The normal-frequency component was varied using the same pressure and frequency ranges as pure NFJV: driving pressures of 0.7, 0.9, 1.1, 1.3, 1.5, 1.7, 1.9, and 2.0 bar, and frequencies of 12, 14, 16, 18, 20, 22, and 24 cycles·min^−1^. This generated 56 parameter combinations for paired airway comparison.

Third, superimposed high-frequency jet ventilation with high-frequency component titration was tested and is referred to as SHFJV-HF. In this strategy, the normal-frequency component was kept constant at a driving pressure of 1.1 bar and a frequency of 12 cycles·min^−1^. The high-frequency component was varied at driving pressures of 0.3, 0.5, 0.7, 0.9, 1.1, 1.3, 1.5, 1.7, 1.9, and 2.0 bar, and frequencies of 60, 300, 600, 900, 1,200, and 1,500 cycles·min^−1^. This generated 60 parameter combinations for paired airway comparison.

For each strategy, identical parameter combinations were tested in both the non-stenotic and 90% stenotic airway models.

### Data acquisition and preprocessing

After each parameter adjustment, the system was allowed to stabilize for 3–5 min. Flow, pressure, tidal volume, PEEP, and FiO_2_ were recorded from the flow analyzer over three respiratory cycles and averaged for each parameter setting and airway condition.

Expiratory flow was displayed as a negative value according to the analyzer convention. Therefore, expiratory flow magnitude was calculated as the absolute value of expiratory flow. Measured distal FiO_2_ represented the oxygen concentration of the mixed gas entering the artificial test lung and was not interpreted as arterial oxygenation, oxygen uptake, or clinical oxygenation.

### Paired airway comparison

For each ventilation strategy, identical parameter combinations under the normal airway and 90% stenotic airway conditions were matched one-to-one. Paired differences were calculated as: Δvalue = value(stenotic airway) − value(normal airway).

Oxygen-ventilation discordance was defined as an increase in measured distal FiO_2_ accompanied by a decrease in VT. Strict discordance was defined as simultaneous increase in FiO_2_, decrease in VT, increase in PEEP, and decrease in expiratory flow magnitude.

To explore whether changes in measured distal FiO_2_ reflected altered gas composition, a simplified two-source mixing model was used. The delivered tidal volume was assumed to consist of oxygen-source gas from the jet source with an oxygen fraction of 1.0 and entrained ambient air with an oxygen fraction of 0.21. Estimated entrained-air fraction was calculated as: (1 − FiO_2_)/0.79. Estimated oxygen-source fraction was calculated as (FiO_2_-0.21)/0.79. Component volumes were obtained by multiplying each fraction by VT before calculating descriptive summaries or paired differences. These were model-derived quantities rather than direct measurements of entrained-air or jet-source flow.

### Statistical analysis and visualization

Continuous variables are summarized as median (Q1 to Q3) across tested settings. Separate linear mixed-effects models were fitted for FiO_2_, VT, PEEP, inspiratory flow, and expiratory flow magnitude. Fixed effects were ventilation strategy, airway condition, and their interaction; a random intercept identified each strategy-specific parameter setting. Models were estimated by restricted maximum likelihood. The strategy × airway interaction was evaluated with a two-degree-of-freedom Wald test. Pairwise contrasts compared the stenosis effect between strategies, with Holm correction across the three contrasts for each outcome. Estimated gas-component volumes were analyzed in the same framework as secondary outcomes. Supportive within-strategy airway comparisons used two-sided Wilcoxon signed-rank tests.

A separate 2 × 2 mixed-effects model compared pure NFJV with SHFJV-NF across the 56 shared NF settings. Fixed effects were airway condition, HF addition, and their interaction, with a random intercept for the shared NF setting. The interaction estimate was ΔΔ = ΔSHFJV-NF − ΔNFJV and was reported with a 95% confidence interval. Strategy-specific response-surface regressions modeled paired changes using standardized component pressure, standardized component frequency, their product, and both quadratic terms; HC3 heteroscedasticity-consistent standard errors were used. Analyses used Python, statsmodels, and SciPy. Two-sided *P* < 0.05 was the nominal significance threshold. Multiplicity correction was applied within each pairwise contrast family, not across outcomes. Inference was exploratory and conditional on the tested parameter grids; parameter settings did not represent independent biological or apparatus replicates.

## Results

### Paired changes induced by 90% airway stenosis

The analysis included 172 paired settings, comprising 56 for pure NFJV, 56 for SHFJV-NF, and 60 for SHFJV-HF. Stenosis increased distal FiO_2_ and measured PEEP and reduced VT, inspiratory flow, and expiratory flow magnitude within each strategy (all Wilcoxon *P* < 0.001). Strategy × airway interactions were significant for all five measured outcomes (all *P* < 0.001; Table 1 and Figure 2).

**Table 1:** Paired changes induced by 90% airway stenosis.

Outcome	Pure NFJV	SHFJV-NF	SHFJV-HF	Strategy × airway *P*
	*n* = 56	*n* = 56	*n* = 60	
ΔFiO_2_, percentage points	4.7 (3.9 to 5.5)^a^	29.1 (27.4 to 29.9)^b^	24.7 (20.2 to 25.9)^c^	<0.001
ΔVT, mL	−454 (−592 to −298)^a^	−577 (−716 to −437)^b^	−232 (−279 to −210)^c^	<0.001
ΔPEEP, cmH_2_O	2.3 (1.0 to 3.5)^a^	4.2 (3.5 to 5.0)^b^	3.2 (2.6 to 5.8)^b^	<0.001
ΔInspiratory flow, L·min^−1^	−33.0 (−40.3 to −26.2)^a^	−48.7 (−55.5 to −42.3)^b^	−30.7 (−38.2 to −28.2)^a^	<0.001
ΔExpiratory flow magnitude, L·min^−1^	−56.7 (−61.9 to −47.9)^a^	−65.9 (−71.5 to −57.2)^b^	−42.5 (−50.5 to −39.6)^c^	<0.001

The superscript letters a-c in Table 1 indicate Holm-adjusted pairwise comparisons of stenosis-induced changes among the three ventilation strategies within each outcome row. Different letters indicate statistically significant differences, whereas shared letters indicate no significant difference. For ΔFiO₂, ΔVT, and Δexpiratory flow magnitude, all three pairwise comparisons were significant (all adjusted *P* < 0.001).

For ΔPEEP, pure NFJV (a) differed significantly from both SHFJV-NF and SHFJV-HF (b), while the two SHFJV strategies did not differ significantly (adjusted *P* = 0.403). For Δinspiratory flow, SHFJV-NF (b) differed significantly from pure NFJV and SHFJV-HF (a), whereas pure NFJV and SHFJV-HF did not differ significantly (adjusted *P* = 0.303).

**Figure 2:**
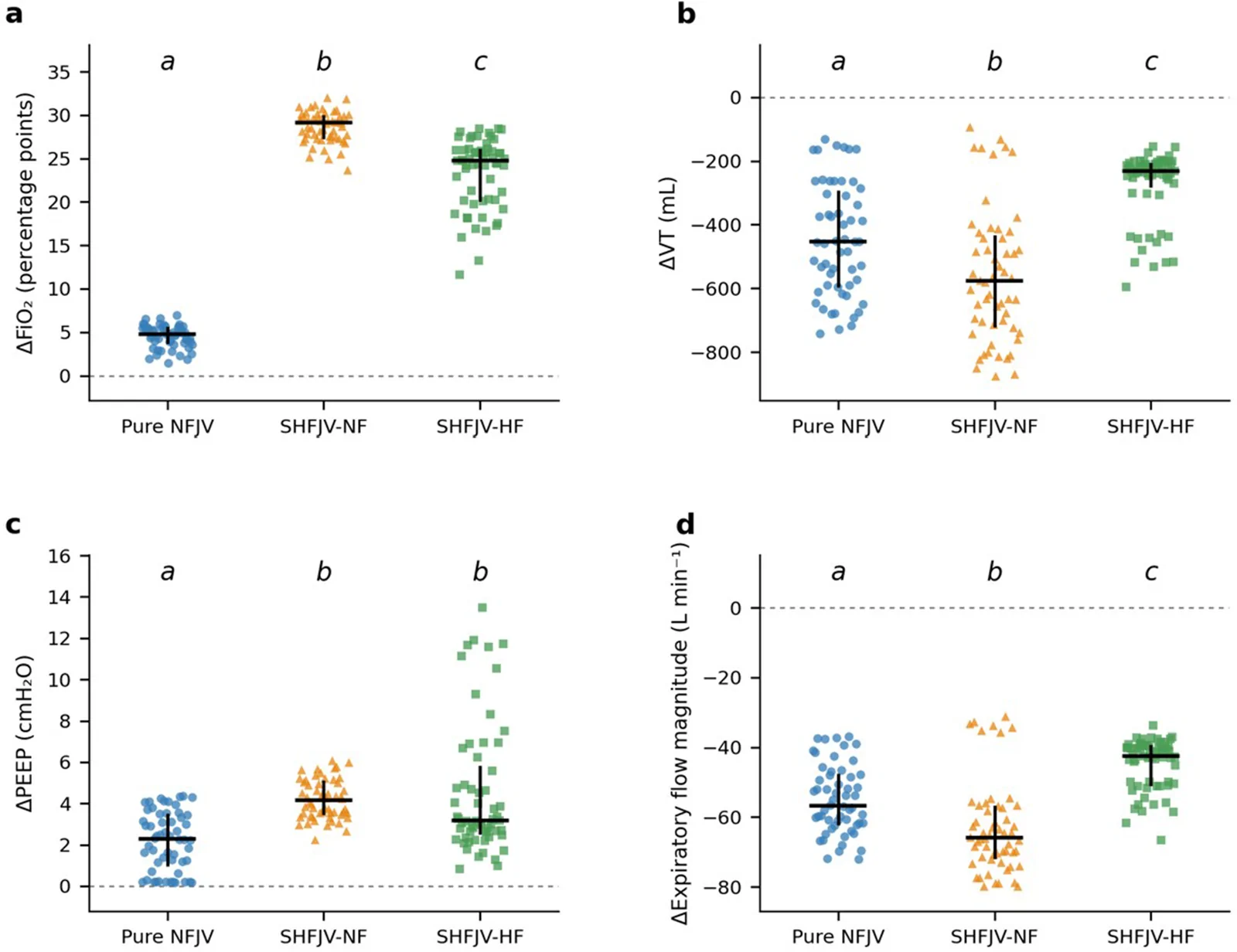
Strategy-specific changes induced by 90% airway stenosis. Points show setting-level paired changes, calculated as 90% stenosis minus non-stenotic airway, for **(a)** FiO_2_, **(b)** VT, **(c)** measured PEEP, and **(d)** expiratory flow magnitude. There are 56 paired settings for pure NFJV, 56 for SHFJV-NF, and 60 for SHFJV-HF. Horizontal black lines show medians, vertical black bars show interquartile ranges, and dashed lines mark zero. Strategy × airway interaction *P* < 0.001 in every panel, from a linear mixed-effects model with a strategy-specific setting random intercept. Bold lowercase letters at the upper left identify the subpanels. Italic lowercase letters above the strategy groups denote Holm-adjusted pairwise interaction contrasts within each subpanel, not fixed ventilation-mode labels. Different statistical letters indicate a significant difference (adjusted *P* < 0.05); groups sharing a letter did not differ significantly. In panels a, b, and d, all pairwise adjusted *P* < 0.001. In panel c, pure NFJV **(a)** differed from SHFJV-NF **(b)** and SHFJV-HF **(b)** (both adjusted *P* < 0.001), whereas SHFJV-NF and SHFJV-HF did not differ significantly (adjusted *P* = 0.403). Within-strategy airway comparisons used two-sided Wilcoxon signed-rank tests (all *P* < 0.001). Settings represent experimental parameter combinations, not independent biological replicates.

Median ΔFiO_2_ was +4.7, +29.1, and +24.7 percentage points for pure NFJV, SHFJV-NF, and SHFJV-HF, respectively. Corresponding median ΔVT values were −454, −577, and −232 mL, and median Δexpiratory flow magnitude values were −56.7, −65.9, and −42.5 L·min^−1^. All three pairwise interaction contrasts were significant for these outcomes after Holm correction (all adjusted *P* < 0.001).

Median ΔPEEP was +2.3, +4.2, and +3.2 cmH_2_O, respectively. Each SHFJV strategy differed from pure NFJV (both adjusted *P* < 0.001), while the SHFJV-NF vs. SHFJV-HF contrast was not significant (adjusted *P* = 0.403). For inspiratory flow, SHFJV-NF differed from both other strategies (both adjusted *P* < 0.001); pure NFJV and SHFJV-HF did not differ significantly (adjusted *P* = 0.303). Full interaction contrasts are provided in Supplementary Table S1.

Increased FiO_2_ with decreased VT occurred in all 172 paired settings (Supplementary Figure S1).

### Matched effect of HF addition and parameter-dependent responses

Across the 56 shared NF settings, HF addition changed the stenosis response by +24.1 percentage points for FiO_2_ (95% CI: 23.4 to 24.8), −111.7 mL for VT (−173.6 to −49.8), +2.02 cmH_2_O for PEEP (1.64 to 2.40), −15.0 L·min^−1^ for inspiratory flow (−18.4 to −11.6), and −8.11 L·min^−1^ for expiratory flow magnitude (−11.72 to −4.50; all *P* < 0.001; Table 2 and Figure 3).

**Table 2:** Matched effect of adding a fixed HF component.

Outcome	Pure NFJV Δ Median (Q1 to Q3)	SHFJV-NF Δ Median (Q1 to Q3)	Model ΔΔ estimate 95% CI	*P*
ΔFiO_2_, percentage points	4.7 (3.9 to 5.5)	29.1 (27.4 to 29.9)	24.1 (23.4 to 24.8)	<0.001
ΔVT, mL	−454 (−592 to −298)	−577 (−716 to −437)	−111.7 (−173.6 to −49.8)	<0.001
ΔPEEP, cmH_2_O	2.3 (1.0 to 3.5)	4.2 (3.5 to 5.0)	2.02 (1.64 to 2.40)	<0.001
ΔInspiratory flow, L·min^−1^	−33.0 (−40.3 to −26.2)	−48.7 (−55.5 to −42.3)	−15.0 (−18.4 to −11.6)	<0.001
ΔExpiratory flow magnitude, L·min^−1^	−56.7 (−61.9 to −47.9)	−65.9 (−71.5 to −57.2)	−8.11 (−11.72 to −4.50)	<0.001

The same 56 NF pressure-frequency settings were compared under both strategies and both airway conditions. The fixed HF component was 0.6 bar at 600 cycles·min^−1^. ΔΔ is the airway × HF-addition interaction from a 2 × 2 linear mixed-effects model with shared NF setting as a random intercept, calculated as the SHFJV-NF stenosis effect minus the pure-NFJV stenosis effect. It is a model-estimated mean contrast, not the difference between the displayed medians. *P* values are two-sided Wald tests and are not multiplicity-adjusted. The effect describes changes from baseline, not a comparison of absolute responses under stenosis.

**Figure 3:**
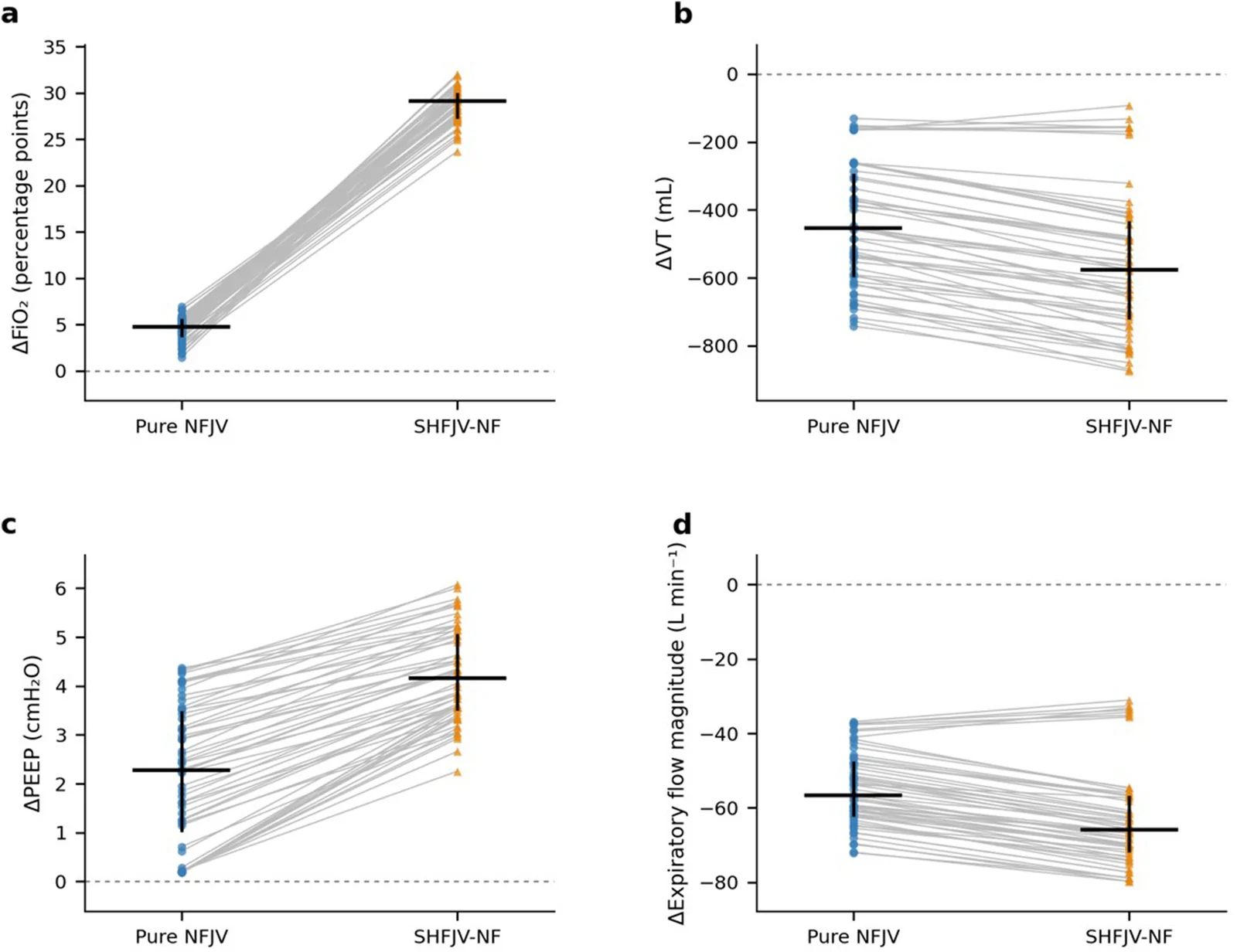
Effect of HF addition at identical NF settings. Each gray line connects stenosis-induced changes at the same NF pressure-frequency setting during pure NFJV and SHFJV-NF (56 matched settings). The fixed HF component was 0.6 bar at 600 cycles·min^−1^. Panels show **(a)** ΔFiO_2_, **(b)** ΔVT, **(c)** ΔPEEP, and **(d)** Δexpiratory flow magnitude. Horizontal black lines indicate medians and vertical black bars indicate interquartile ranges. Dashed lines mark zero change. HF addition × airway interaction *P* < 0.001 for all panels in a 2 × 2 linear mixed-effects model with shared NF setting as the random-intercept grouping factor. Difference-in-differences estimates and 95% confidence intervals are reported in Table 2. These contrasts concern changes with stenosis, not absolute performance under stenosis.

Strategy-specific pressure-frequency models were significant for all five measured outcomes (all global *P* < 0.001), with R² values from 0.428 to 0.991 (Supplementary Table S2). In SHFJV-HF, VT changes were associated with the linear and quadratic pressure terms (both *P* < 0.001), while the linear frequency term was not significant (*P* = 0.818). FiO_2_, PEEP, and both flow outcomes showed significant pressure and frequency terms in this strategy.

### Estimated gas composition

Estimated entrained-air fractions in the non-stenotic airway were 33.5% for pure NFJV, 42.3% for SHFJV-NF, and 38.8% for SHFJV-HF. With stenosis, these fractions were 27.7%, 5.6%, and 7.8%, respectively (Table 3).

**Table 3:** Estimated gas fractions across airway conditions.

Airway condition	Strategy	FiO_2_, %	VT, mL	Estimated air fraction, %	Estimated oxygen-source fraction, %
Non-stenotic	Pure NFJV	73.5 (72.4 to 74.6)	1,238.2 (942.4 to 1,487.5)	33.5 (32.1 to 34.9)	66.5 (65.1 to 67.9)
Non-stenotic	SHFJV-NF	66.6 (65.6 to 67.6)	1,312.6 (1,090.1 to 1,535.8)	42.3 (41.0 to 43.5)	57.7 (56.5 to 59.0)
Non-stenotic	SHFJV-HF	69.4 (67.6 to 70.7)	842.5 (754.4 to 912.0)	38.8 (37.0 to 41.0)	61.2 (59.0 to 63.0)
90% stenosis	Pure NFJV	78.1 (77.4 to 79.1)	734.9 (595.3 to 918.4)	27.7 (26.4 to 28.5)	72.3 (71.5 to 73.6)
90% stenosis	SHFJV-NF	95.6 (95.1 to 95.7)	695.8 (603.9 to 853.2)	5.6 (5.4 to 6.2)	94.4 (93.8 to 94.6)
90% stenosis	SHFJV-HF	93.8 (91.1 to 94.8)	594.7 (524.3 to 641.3)	7.8 (6.6 to 11.2)	92.2 (88.8 to 93.4)

Values are median (Q1 to Q3) across 56, 56, and 60 settings for pure NFJV, SHFJV-NF, and SHFJV-HF, respectively. Fractions were calculated separately at each setting and airway condition using air fraction = (1 − FiO_2_)/0.79 and oxygen-source fraction = (FiO_2_ − 0.21)/0.79, with FiO_2_ as a decimal. Fractions sum to 100% at each setting. Fractions and volumes are inferred under a two-source mixing assumption, not measured source flows. Paired changes in estimated component volumes are shown in Figure 4; pairwise interaction contrasts are provided in Supplementary Table S1. Separate medians of component volumes need not sum to the median VT, and a median paired change need not equal the difference of marginal medians.

Median paired changes in estimated entrained-air volume were −197 mL (Q1 to Q3, −236 to −141), −491 mL (−605 to −418), and −288 mL (−307 to −255), respectively. The strategy × airway interaction and all Holm-adjusted pairwise interaction contrasts were significant (all *P* < 0.001; Figure 4 and Supplementary Table S1).

**Figure 4:**
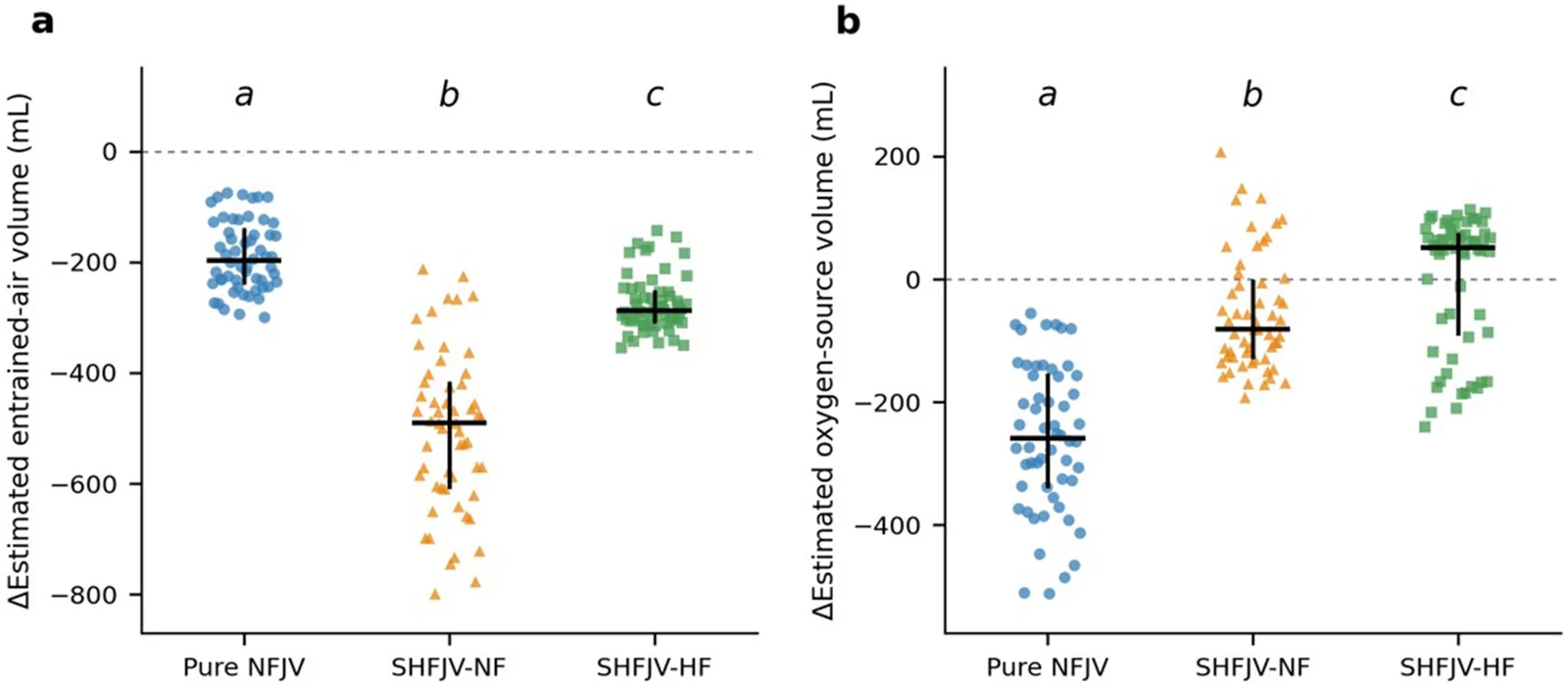
Paired changes in estimated gas-component volumes. Panels show **(a)** estimated entrained-air volume and **(b)** estimated oxygen-source volume. The two-source mixing calculation was applied to each individual setting and airway condition before paired changes were calculated. All 56 pure-NFJV, 56 SHFJV-NF, and 60 SHFJV-HF pairs are shown. Horizontal black lines indicate medians; vertical bars indicate interquartile ranges; dashed lines mark zero. Strategy × airway interaction *P* < 0.001 in each panel. Within each panel, italic lowercase letters above the strategy groups indicate significant pairwise interaction contrasts after Holm correction [all adjusted *P* < 0.001 in panel **(a)**]; all adjusted *P* < 0.01 in panel **(b)**. Component estimates are derived from FiO_2_ and VT and are not direct measurements of entrained-air or jet-source flow.

## Discussion

Severe fixed airway stenosis produced a consistent oxygen–ventilation discordance in this open rigid-bronchoscopy bench model: distal FiO_2_ increased, whereas VT and expiratory flow magnitude decreased at every one of the 172 paired parameter settings. However, the magnitude of this response differed among pure NFJV, SHFJV-NF, and SHFJV-HF. At the 56 identical NF pressure–frequency settings, adding a fixed HF component amplified the stenosis-associated FiO_2_ increase, VT loss, expiratory-flow reduction, and measured PEEP increase. These findings indicate that a higher distal oxygen concentration under stenosis does not establish preserved total gas delivery and that the response to narrowing depends on the applied jet-ventilation configuration. Earlier single-frequency bench work similarly found increased oxygen concentration alongside reduced volume delivery under severe stenosis (7).

Gas delivery during rigid-bronchoscopy jet ventilation occurs through an open system, in which oxygen from the jet source mixes with ambient air admitted through the proximal openings and the space around the bronchoscope sheath (1, 2). The fraction of ambient air reaching the distal measurement site therefore influences the measured FiO_2_. Importantly, stenosis does not have a uniform effect on jet-induced entrainment. Buczkowski et al. found greater entrainment, distal pressure, and minute volume when the jet was delivered above rather than through or below a stenosis; the responses also depended on stenosis diameter (4). Other model studies demonstrated that injector position relative to an obstruction changes pressure transmission (5, 6). These findings establish the importance of geometry and jet location, rather than a general rule that narrowing must always reduce entrainment. In the present proximal-jet, open-sheath configuration, the increased distal FiO_2_ was accompanied by a smaller model-estimated ambient-air contribution.

The setting-level gas-composition analysis clarifies why oxygen concentration and total delivered volume moved in opposite directions. The estimated ambient-air contribution decreased, most markedly during SHFJV-NF. Although the oxygen-source fraction necessarily increased, the accompanying VT reduction meant that oxygen-source volume did not increase consistently. A more oxygen-rich distal mixture therefore should not be equated with greater absolute oxygen-source volume or total volume delivery. These estimates were calculated from FiO_2_ and VT, not independent measurements of nozzle output or ambient-air flow. They are consistent with reduced oxygen dilution but cannot establish whether reduced entrainment, recirculation, or incomplete mixing accounted for the observed pattern.

The accompanying reductions in VT and expiratory flow place the composition findings in a broader mechanical context. Experimental studies of stenotic jet ventilation have shown that injector position and obstruction geometry can alter distal pressure and gas delivery (4–6), while our previous single-frequency bench study documented reduced volume and expiratory flow with severe narrowing (7). Increased resistance and insufficient expiratory time can promote incomplete emptying and gas trapping during high-frequency jet ventilation; these effects have been demonstrated in test-lung experiments, particularly with higher driving pressure or prolonged inspiration (11). End-expiratory pressure has also been assessed during transtracheal jet ventilation as a means of detecting pressure accumulation (12). The higher analyzer-recorded PEEP in the present study is compatible with altered pressure dissipation and expiratory emptying, but it is not a direct measurement of regional alveolar intrinsic PEEP, trapped gas volume, or lung hyperinflation. Reduced exchange with the ambient-air pathway and restricted expiratory egress remain mechanistic interpretations of our open model, not independently measured flow paths.

The matched comparison helps separate the effect of HF addition from differences in the sampled NF settings. At identical NF settings, HF addition amplified the stenosis-associated changes in FiO_2_, VT, measured PEEP, and expiratory flow magnitude (Table 2). The larger non-stenotic estimated ambient-air component during SHFJV-NF and its pronounced decline after stenosis offer a compositional explanation for the marked FiO_2_ response. Earlier lung-simulator work by Aloy et al. described greater inspiratory gas entrainment and VT, together with altered expiratory flow and end-expiratory pressure, when low- and high-frequency jet streams were superimposed (13). That study supplies a plausible physical context, but differences in jet position, airway anatomy, and obstruction mean that its results cannot be assumed to apply unchanged to the present system. The present matched findings identify how HF addition modifies the response to stenosis; they do not demonstrate improved effective ventilation or CO_2_ clearance.

The two SHFJV strategies addressed different parameter questions. In SHFJV-NF, HF settings were held constant while NF pressure and frequency varied; in SHFJV-HF, NF settings were fixed while HF pressure and frequency varied. Consequently, their aggregate medians reflect both the ventilation configuration and the particular parameter grid, and the smaller median stenosis-induced VT decrement during SHFJV-HF cannot be interpreted as superior absolute volume delivery. Its median VT under stenosis was lower than that of the other two strategies. Strategy-specific response-surface analyses further indicated pressure- and frequency-dependent, sometimes nonlinear responses. In the SHFJV-HF VT model, pressure terms were significant whereas the linear HF-frequency term was not (*P* = 0.818); this finding is conditional on the tested settings and model specification and does not establish that HF frequency is physiologically irrelevant. Frequency-dependent effects on end-expiratory volume, VT, and gas exchange have been documented in an unobstructed porcine comparison of SHFJV and HFJV (14), but those data do not define the response in a severely stenotic open rigid-bronchoscopy system.

Prior clinical and animal studies provide complementary rather than directly interchangeable evidence. SHFJV has been used during endoscopic laryngotracheal procedures (8), and Leiter et al. compared SHFJV with several single-frequency techniques in patients undergoing minor laryngeal surgery (9). In a porcine tracheal-obstruction model, Sütterlin et al. compared SHFJV with single-frequency HFJV and observed frequency- and obstruction-dependent changes in lung volume and gas exchange, including hypercapnia with the narrowest stent (10). However, that study did not include pure NFJV and did not isolate HF addition at identical NF settings. Our experiment addresses this component-specific comparison in a controlled bench system, whereas those *in vivo* studies evaluated physiological gas exchange; their endpoints should not be treated as equivalent.

The interpretation of distal FiO_2_ has an important practical boundary. It represents the oxygen concentration of mixed gas entering the test lung, not arterial oxygenation, oxygen uptake, or CO_2_ elimination. Previous physiological work established that CO_2_ clearance during high-frequency jet ventilation depends on effective gas delivery and respiratory mechanics (15); clinical work during rigid bronchoscopy likewise supports direct CO_2_ monitoring rather than relying on oxygenation alone (16). In this study, every paired setting showed higher distal FiO_2_ alongside lower VT, and many settings also showed greater measured PEEP. Accordingly, distal oxygen concentration needs to be considered alongside volume delivery, inspiratory and expiratory flow, and pressure. Whether these bench patterns translate into adequate PaO_2_ or PaCO_2_ in patients cannot be determined from the present measurements.

Several limitations constrain interpretation. This was a fixed *in vitro* model without pulmonary perfusion, oxygen uptake, CO_2_ production, heterogeneous regional mechanics, spontaneous effort, or hemodynamic responses. Only one fixed stenosis severity was examined; irregular stenosis, moderate narrowing, secretions, and dynamic airway collapse were not modeled. The three-strategy analysis used unequal and non-equivalent parameter grids, although the separate 56-setting comparison controlled the NF grid when assessing HF addition. Parameter settings were repeated conditions within one experimental platform, not independent biological or apparatus replicates; model-based confidence intervals and *P* values should therefore be interpreted within that design. The two-source composition model assumes complete mixing of a pure-oxygen jet source with ambient air, and the inferred component volumes are not direct flow measurements. Ventilator-displayed driving pressure was not nozzle exit pressure, and analyzer-recorded PEEP was not alveolar intrinsic PEEP. Neither arterial blood gases nor CO_2_ elimination were measured. These results characterize strategy-dependent gas delivery in the tested setup, but do not define a safe clinical parameter range or the clinical superiority of any strategy.

## Conclusion

In an open rigid-bronchoscopy bench model, adding a fixed HF component at identical NF settings amplified the stenosis-associated rise in distal FiO_2_ and the reductions in VT and expiratory flow, while increasing the PEEP response. Titrating the HF component at fixed NF settings produced a distinct response across its tested parameter range. Estimated loss of ambient-air contribution was consistent with reduced oxygen dilution, but was not directly measured. These findings define strategy-dependent gas-delivery behavior under severe stenosis; they do not establish preserved arterial oxygenation, CO_2_ clearance, or clinical superiority of a ventilation strategy.

## Data Availability

The raw data supporting the conclusions of this article will be made available by the authors, without undue reservation.
