# Supplementary material for: Airway stenosis alters the balance between tidal volume, air entrainment, and oxygen-source delivery during normal frequency and superimposed jet ventilation: a bench model study
Source: Front Med (Lausanne). 2026 Oct 28;13:1910316. doi: 10.3389/fmed.2026.1910316 (PMC13751342; doi:10.3389/fmed.2026.1910316)
Supplement: Supplementary file 1 [file Table1.docx]

**Supplementary Table S1 Pairwise contrasts of the stenosis effect**

| **Outcome** | **Difference in stenosis effect** | **Model estimate (95% CI)** | **Holm-adjusted P** |
| --- | --- | --- | --- |
| ΔFiO₂, percentage points | SHFJV-NF - Pure NFJV | 24.12 (23.18 to 25.06) | <0.001 |
| ΔFiO₂, percentage points | SHFJV-HF - Pure NFJV | 18.81 (17.88 to 19.73) | <0.001 |
| ΔFiO₂, percentage points | SHFJV-NF - SHFJV-HF | 5.31 (4.39 to 6.24) | <0.001 |
| ΔVT, mL | SHFJV-NF - Pure NFJV | -111.72 (-174.59 to -48.84) | <0.001 |
| ΔVT, mL | SHFJV-HF - Pure NFJV | 170.28 (108.46 to 232.09) | <0.001 |
| ΔVT, mL | SHFJV-NF - SHFJV-HF | -281.99 (-343.81 to -220.18) | <0.001 |
| ΔPEEP, cmH₂O | SHFJV-NF - Pure NFJV | 2.02 (1.23 to 2.81) | <0.001 |
| ΔPEEP, cmH₂O | SHFJV-HF - Pure NFJV | 2.35 (1.58 to 3.12) | <0.001 |
| ΔPEEP, cmH₂O | SHFJV-NF - SHFJV-HF | -0.33 (-1.10 to 0.44) | 0.403 |
| ΔInspiratory flow, L·min⁻¹ | SHFJV-NF - Pure NFJV | -15.01 (-18.61 to -11.41) | <0.001 |
| ΔInspiratory flow, L·min⁻¹ | SHFJV-HF - Pure NFJV | -1.86 (-5.40 to 1.68) | 0.303 |
| ΔInspiratory flow, L·min⁻¹ | SHFJV-NF - SHFJV-HF | -13.15 (-16.69 to -9.61) | <0.001 |
| ΔExpiratory flow magnitude, L·min⁻¹ | SHFJV-NF - Pure NFJV | -8.11 (-11.93 to -4.29) | <0.001 |
| ΔExpiratory flow magnitude, L·min⁻¹ | SHFJV-HF - Pure NFJV | 10.07 (6.31 to 13.82) | <0.001 |
| ΔExpiratory flow magnitude, L·min⁻¹ | SHFJV-NF - SHFJV-HF | -18.17 (-21.93 to -14.42) | <0.001 |
| Δ estimated entrained-air volume, mL | SHFJV-NF - Pure NFJV | -314.38 (-349.27 to -279.49) | <0.001 |
| Δ estimated entrained-air volume, mL | SHFJV-HF - Pure NFJV | -86.57 (-120.87 to -52.26) | <0.001 |
| Δ estimated entrained-air volume, mL | SHFJV-NF - SHFJV-HF | -227.81 (-262.11 to -193.51) | <0.001 |
| Δ estimated oxygen-source volume, mL | SHFJV-NF - Pure NFJV | 202.66 (162.53 to 242.79) | <0.001 |
| Δ estimated oxygen-source volume, mL | SHFJV-HF - Pure NFJV | 256.84 (217.39 to 296.30) | <0.001 |
| Δ estimated oxygen-source volume, mL | SHFJV-NF - SHFJV-HF | -54.18 (-93.64 to -14.73) | 0.007 |

Contrasts were obtained from the three-strategy linear mixed-effects models. The comparison direction is the first named strategy minus the second. Holm adjustment was applied across three contrasts separately for each outcome. Outcomes are not multiplicity-adjusted as a single family. Estimated gas-component outcomes are secondary and algebraically derived from FiO₂ and VT.

**Supplementary Table S2 Pressure-frequency response-surface models**

| **Strategy** | **Outcome** | **R²** | **Global P** | **Pressure P** | **Frequency P** | **P × F P** | **Pressure² P** | **Frequency² P** |
| --- | --- | --- | --- | --- | --- | --- | --- | --- |
| Pure NFJV | ΔFiO₂ | 0.513 | <0.001 | <0.001 | <0.001 | 0.650 | 0.082 | 0.449 |
| Pure NFJV | ΔVT | 0.974 | <0.001 | <0.001 | <0.001 | 0.131 | 0.028 | <0.001 |
| Pure NFJV | ΔPEEP | 0.952 | <0.001 | <0.001 | <0.001 | 0.362 | 0.799 | <0.001 |
| Pure NFJV | ΔInspiratory flow | 0.988 | <0.001 | <0.001 | 0.117 | 0.369 | <0.001 | 0.013 |
| Pure NFJV | ΔExpiratory flow magnitude | 0.991 | <0.001 | <0.001 | <0.001 | <0.001 | <0.001 | 0.731 |
| SHFJV-NF | ΔFiO₂ | 0.428 | <0.001 | 0.073 | 0.180 | 0.113 | <0.001 | 0.955 |
| SHFJV-NF | ΔVT | 0.971 | <0.001 | <0.001 | 0.855 | 0.006 | <0.001 | <0.001 |
| SHFJV-NF | ΔPEEP | 0.923 | <0.001 | <0.001 | <0.001 | <0.001 | 0.440 | 0.057 |
| SHFJV-NF | ΔInspiratory flow | 0.941 | <0.001 | <0.001 | 0.038 | 0.512 | <0.001 | 0.800 |
| SHFJV-NF | ΔExpiratory flow magnitude | 0.917 | <0.001 | <0.001 | 0.043 | 0.407 | <0.001 | 0.560 |
| SHFJV-HF | ΔFiO₂ | 0.679 | <0.001 | <0.001 | 0.003 | 0.104 | <0.001 | <0.001 |
| SHFJV-HF | ΔVT | 0.749 | <0.001 | <0.001 | 0.818 | 0.420 | <0.001 | 0.432 |
| SHFJV-HF | ΔPEEP | 0.927 | <0.001 | <0.001 | <0.001 | 0.019 | 0.016 | <0.001 |
| SHFJV-HF | ΔInspiratory flow | 0.743 | <0.001 | <0.001 | <0.001 | 0.305 | <0.001 | <0.001 |
| SHFJV-HF | ΔExpiratory flow magnitude | 0.619 | <0.001 | <0.001 | <0.001 | 0.913 | <0.001 | 0.028 |

Each model used the setting-level paired stenosis change as the dependent variable and standardized titrated-component pressure, standardized frequency, their product, and both quadratic terms as predictors. HC3 heteroscedasticity-consistent standard errors were used. Pressure and frequency are NF settings for pure NFJV and SHFJV-NF and HF settings for SHFJV-HF. P values are exploratory, unadjusted term or global tests. These models describe variation across the tested grids and do not imply biological replication.
