# Supplementary material for: Airway stenosis alters the balance between tidal volume, air entrainment, and oxygen-source delivery during normal frequency and superimposed jet ventilation: a bench model study
Source: Front Med (Lausanne). 2026 Oct 28;13:1910316. doi: 10.3389/fmed.2026.1910316 (PMC13751342; doi:10.3389/fmed.2026.1910316)
Supplement: Supplementary file 2 [file Table2.docx]

**Supplementary Figure S1 FiO₂–VT discordance induced by stenosis**

Each point shows the paired change in FiO₂ and VT at one identical parameter setting between airway conditions. All 172 pairs had ΔFiO₂>0 and ΔVT<0, comprising 56 pure-NFJV, 56 SHFJV-NF, and 60 SHFJV-HF settings. Dashed horizontal and vertical lines mark zero change. The plot is descriptive and retains all original paired observations.
